# Effects of herbal medicine for dysmenorrhea treatment on accompanied acne vulgaris: a study protocol for a randomized controlled trial

**DOI:** 10.1186/s12906-017-1813-1

**Published:** 2017-06-17

**Authors:** Kwan-Il Kim, Hae Jeong Nam, Mia Kim, Junhee Lee, Kyuseok Kim

**Affiliations:** 1 Department of Clinical Korean Medicine, College of Korean Medicine, Kyung Hee University, 23 Kyungheedae-ro, Dongdaemun-gu, Seoul, 02447 Republic of Korea; 2 Department of Ophthalmology & Otolaryngology & Dermatology of Korean Medicine, College of Korean Medicine, Kyung Hee University, 23 Kyungheedae-ro, Dongdaemun-gu, Seoul, 02447 Republic of Korea; 3 Department of Cardiovascular and Neurologic disease (Stroke center), College of Korean Medicine, Kyung Hee University, 23 Kyungheedae-ro, Dongdaemun-gu, Seoul, 02447 Republic of Korea; 4 Department of Sasang Consitutional Medicine, College of Korean Medicine, Kyung Hee University, 23 Kyungheedae-ro, Dongdaemun-gu, Seoul, 02447 Republic of Korea; 5 Korean Medicine Clinical Trial Center, Kyung Hee University Korean Medicine Hospital, 23 Kyungheedae-ro, Dongdaemun-gu, Seoul, 02447 Republic of Korea

**Keywords:** Acne vulgaris, Dysmenorrhea, Herbal medicine, *Gyejibokryung-hwan*, *Dangguijagyag-san*

## Abstract

**Background:**

The incidence of preadolescent acne among women is increasing. Acne deteriorates the quality of life; conventional treatment options are limited and have not been effective against acne, particularly acne associated with menstruation. Despite evidence that acne associated with menstruation abnormalities naturally improves when menstruation recovers to normal, there have only been few studies on the effects of dysmenorrhea treatment on acne. Therefore- we designed this study to assess the effects of gyejibokryung-hwan (GBH) and dangguijagyag-san (DJS), which are widely used in dysmenorrhea treatment, on acne associated with menstruation cycle.

**Methods:**

This is a protocol for a randomized, double-blind, parallel-group, placebo-controlled and multicenter trial. One hundred and sixteen participants with dysmenorrhea accompanied by acne vulgaris will be recruited at three centers and randomized into two groups, the herbal treatment group and placebo group. The participants will receive GBH or DJS based on pattern identification or placebo granules thrice daily for 8 weeks, with an 8-week follow up. The primary outcome will be the mean percentage change in the count of inflammatory acne lesions. The secondary outcomes would be based on dysmenorrhea numeric rating scale, verbal multidimensional scoring system for dysmenorrhea, acne numeric rating scale, investigator’s static global assessment scale of facial acne vulgaris, and safety testing. Adverse events will also be reported.

**Discussion:**

The effects of GBH or DJS used in dysmenorrhea treatment on acne associated with the menstrual cycle will be evaluated. The findings of this trial will provide evidence regarding the effect of herbal medicine in improving acne vulgaris associated with menstruation in women.

**Trial registration:**

Korean Clinical Trial Registry (http://cris.nih.go.kr; registration number: KCT0002259). Date of registration: March 10, 2017

## Background

Acne is a chronic inflammatory skin disease of the pilosebaceous unit. The primary causes of acne include increased sebum secretion, follicular hyperkeratinization, *Propionibacterium acnes* within the follicle, and inflammatory responses [1]. Acne is considered an adolescent affection, but over the last few years, adult acne have been increased [2–4]. Postadolescent acne appears to occur more frequently in women than in men [5, 6]. The unique features of adult acne in women are that it is related to the menstrual cycle [3, 7]. Up to 78% of women with acne have premenstrual flares [3]. Accumulation of sebum within the sebaceous glands due to changes in sex hormone secretion during ovulation and menstruation has been proposed as a potential contributor to premenstrual acne [8, 9]; however, the mechanism underlying this phenomenon remains unknown.

Because adult acne in women is associated with menstrual-related hormones, it is usually accompanied by menstrual abnormalities, i.e. dysmenorrhea. Primary dysmenorrhea is defined as pain during menstruation in the absence of pelvic problems [10–12]. The prevalence of dysmenorrhea varies from 16 to 83% in women of reproductive age [10, 13]. Oral contraceptives are used to treat dysmenorrhea [11] and administered to female patients with acne who do not respond to medication and are suspected of having hormonal abnormalities [14, 15]. But the actual frequency of prescribing oral contraceptives is low because of possible side effects such as nausea, vomiting, and weight gain and social conventions against single female patients consuming oral contraceptives.

In traditional Chinese medicine and traditional Korean medicine (TKM), blood stagnation during ovulation and menstruation is considered the cause of dysmenorrhea [16, 17] and aggravated acne associated with the menstrual cycle. Therefore, treatment with herbal medicine for dysmenorrhea often improves symptoms of acne associated with the menstrual cycle; however, no study on this has been conducted thus far. Gyejibokryung-hwan (GBH) and dangguijagyag-san (DJS), administered in this trial, have been long used in East Asian countries for treatment of dysmenorrhea [16, 18, 19]. The effects of GBH and DJS on dysmenorrhea have been investigated [16, 18, 19]. The prescription of the two herbal medicines is usually determined by a physical state diagnosis according to TKM patterns [16, 17]. Excess-deficiency pattern for dysmenorrhea is importantly considered. In general, GBH is used for the excess pattern of dysmenorrhea and DJS for deficiency pattern of dysmenorrhea. Administration based on the pattern improves clinical results; therefore, we aimed to reflect the clinical condition to the research.

The objective of this trial was to determine whether the herbal medicines prescribed to treat dysmenorrhoea based on the pattern could simultaneously improve acne vulgaris associated with the menstrual cycle.

## Methods / Design

### Objective

To investigate the effects of GBH and DJS on acne vulgaris in patients who were administered these herbal medicines for dysmenorrheal treatment.

### Hypothesis

The primary objective is to investigate whether herbal medicine treatment for dysmenorrhea can reduce the number of acne vulgaris lesions in comparison with that reported with placebo treatment. The primary null hypothesis is as follows: Eight-week GBH and DJS treatment does result in any change in the number of acne vulgaris lesions between the treatment group and placebo group.

### Design

**Fig. 1:**
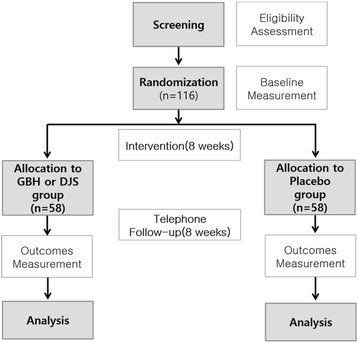
Study flow chart

This study is a randomized, placebo-controlled, double-blind, parallel-group and multicenter trial. The flow chart of the study has been presented in Fig. 1.

### Ethics

The protocol design is in accordance with the Declaration of Helsinki and Korean Good Clinical Practice Guidelines. This trial is authorized by the Institutional Review Board of the Kyung Hee University Korean Medicine Hospital (approval number: KOMCIRB-161014-HR-053), Kyung Hee University Korean Medicine Hospital at Gangdong (approval number: KHNMCOH2016–10–003-003) and Woosuk University Korean Medicine Medical Center (approval number: WSOH IRB 1703–02). Signed informed consent forms will be obtained from all eligible participants before enrollment. This trial is registered with the Korean Clinical Trial Registry (CRIS), Republic of Korea: KCT0002259.

### Participants

#### Diagnostic criteria for excess and deficiency patterns in TKM

A doctor practicing Korean medicine will judge excess and deficiency patterns of dysmenorrhea based on chief physical signs: color of lower eyelid, facial complexion, tongue diagnosis, abdominal examination, and hot flush [20].

Deficiency patterns of dysmenorrhea may comprise pale lower eyelid, pale white complexion, pale tongue, weak abdominal tension and not existing or slightly existing of the feeling of heat up, respectively. Excess patterns of dysmenorrhea may comprise normal or red colored lower eyelid, darkish complexion, red tongue, tight and strong abdominal tension, presence of hot flush.

#### Inclusion criteria

1. Women older than 19 years and of childbearing age
2. Patients who have more than 10 inflammatory acne lesions on their face
3. Patients who have menstrual pain score of more than grade 1 according to the verbal multidimensional scoring system (VMSS)
4. Patients who had regular menstrual cycle of 23–33 days in the last 3 months
5. Patients in who acne vulgaris related to the menstrual cycle aggravated
6. Written informed consent

#### Exclusion criteria

1. Patients who consumed the following medicines:
  - Topical or systemic corticosteroids (in the last 1 month)
  - Herbal medicine for acne treatment (in the last 1 month)
  - Nonsteroidal anti-inflammatory drugs (NSAIDs) (in the last 2 weeks)
  - Oral contraceptives (in the last 1 month)
  - Topical or oral vitamin A derivatives (in the last 1 month)
  - Antibiotics (in the last 1 month)
  - Other unsuitable drugs as judged by the investigators
2. Body Mass Index (BMI) <19 or BMI ≥ 25
3. Liver function abnormality [aspartate aminotransferase (AST) or alanine aminotransferase (ALT) ≥ 60 IU/L] or renal function abnormality [blood urea nitrogen (BUN) ≥ 30 mg/dL or a serum creatinine levels ≥1.5 mg/dL]
4. Pregnancy or breastfeeding or planning to conceive [human chorionic gonadotropin (hCG)-positive women]
5. Patients who have malignant tumor, serious infection, or severe systemic diseases
6. Patients who participated in other clinical trials in the last 3 months
7. Unsuitability as judged by the investigators

#### Rejection and withdrawal criteria

1. Use of forbidden drugs, such as topical or systemic corticosteroids, herbal medicine, oral contraceptives, topical or oral vitamin A derivatives and antibiotics
2. Treatment that might influence the results of the trial without the agreement of a researcher
3. Voluntary withdrawal from the trial
4. Participants who have skin rash, flare or itching during the trial
5. Participants not following the protocol, or taking <80% of the prescribed doses
6. Participants who have anorexia, vomiting, nausea or diarrhea after taking the drug

### Recruitment

Through advertisements and referrals, a total of 116 patients with dysmenorrhea accompanied with acne vulgaris will be recruited at the Kyung Hee University Korean Medicine Hospital, Kyung Hee University Korean Medicine Hospital at Gangdong and Woosuk University Korean Medicine Medical Center.

### Randomization and concealment

Random numbers will be generated by stratified block randomization in random block of 4, and the 116 patients will be randomly allocated in a 1:1 ratio to herbal treatment group or the placebo control. An independent statistician who is unaware of the design and purpose of the study will generate a randomization table using SAS software for Microsoft window (version 9.1.3; SAS Institute Inc., NC, Cary, USA). The statistician will keep the randomization lists and inform the researcher of the matching cord number either by text message or via mobile communication application.

Eligible participants will be screened using inclusion/exclusion criteria and the participants who meet the criteria will be included in the trial. After signing the informed consent form, the patients will be randomly allocated to the two groups.

### Blinding

Participants, investigators, and the clinical trial pharmacist will be blinded to the treatment allocation throughout the course of the study. The statistician will uncover the blinding when necessary, such as on occurrence of a serious adverse event (SAE). The researcher has to inform the statistician when the blinding has to be uncovered. After the confirmation and approval about the reason for cord break, the procedure for uncovering the blinding will begin.

### Interventions

#### GBH and DJS

The experimental group will be administered GBH or DJS granules according to the excess or deficiency pattern identification. The excess pattern group for dysmenorrhea will be orally administered GBH granules (3 g/pouch) thrice daily before or between each meal for 8 weeks. The deficiency pattern group for dysmenorrhea will be administered DJS granules (3 g/pouch) thrice daily before or between each meal for 8 weeks.

The GBH and DJS granules were manufactured by Hanpoong Pharm & Food Co. Ltd. (Jeonju, Republic of Korea) in line with Korea Good Manufacturing Practice authorization. Both GBH and DJS were approved by the Korean Ministry of Food and Drug Safety. GBH and DJS contain 3 g (dry weight) of water-extracted granules combined with lactose and starch. GBH is composed of 5 herbs: Cinnamomi Ramulus (*Cinnamomum cassia* Presl, family Lauraceae; 1.33 g), Poria Sclerotium (*Poria cocos* Wolf, family Polyporaceae; 1.33 g), Moutan Radicis Cortex (*Paeonia suffruticosa* Andrews, family Paeoniaceae; 1.33 g), Persicae Semen (*Prunus persica* Batsch or *Prunus davidiana* Franchet, family Rosaceae; 1.33 g), Paeoniae Radix (*Paeonia lactiflora* Pallas, family Paeoniaceae; 1.33 g). DJS is composed of 6 herbs: Angelicae Gigantis Radix (*Angelica gigas* Nakai*,* family Umbelliferae; 1.00 g), Cnidii Rhizoma (*Cnidium officinale* Makino or *Ligusticum chuanxiong* Hort. family Umbelliferae; 1.00 g), Paeoniae Radix (*Paeonia lactiflora* Pallas, family Paeoniaceae; 2.00 g), Poria Sclerotium (*Poria cocos* Wolf, family Polyporaceae; 1.33 g), Atractylodis Rhizoma Alba (*Atractylodes japonica* Koidzumi or *Atractylodes macrocephala* Koidzumi, family Compositae; 1.33 g), Alismatis Rhizoma (*Alisma orientale* Juzepzuk, family Alismataceae; 1.67 g). Voucher specimens will be reserved at the research library of Hanpoong Pharm & Food Company.

#### Placebo

The placebo group will receive the placebo, which does not contain any active ingredients, but is similar in appearance, taste, and aroma to the GBH or DJS granules. Placebo will be orally taken (3 g/pouch) thrice daily before or between each meal for 8 weeks. The excess pattern group for dysmenorrhea will receive the GBH placebo and the deficiency pattern group for dysmenorrhea will receive DJS placebo.

The placebo was also manufactured by Hanpoong Pharm & Food Co. Ltd. (Jeonju, Republic of Korea), following the placebo guidelines of the Korean Ministry of Food and Drug Safety. All products were packaged by Hanpoong Pharm & Food Co. Ltd.. Either 45 GBH or DJS or placebo pouches will be provided to each randomized participant every two weeks, at visit 2 (week 0) to visit 6 (week 8).

#### Co-interventions

If participants experience unbearable pain during menstruation, taking NSAIDs is permitted. The participants will be asked to record whether they take the NSAIDs or not. Other drugs that alleviate menstrual pain or acne- such as topical or systemic corticosteroids, herbal medicine, oral contraceptives, topical or oral vitamin A derivatives and antibiotics- are prohibited.

### Outcomes

The detailed outcome measurement time points are provided in Table 1.

**Table 1:** Study process treatment and outcome measurements

Activity	Visit 1	Visit 2	Visit 3	Visit 4	Visit 5	Visit 6	Visit 7	Visit 8
Time Schedule	Week 1	Baseline	Week 2	Week 4	Week 6	Week 8	Week 12	Week 16
Window visit		±3 days	±3 days	±3 days	±3 days	±3 days	±3 days	±3 days
Demographic information-taking	X							
Informed consent form	X							
General medical history-taking	X							
Photographing the patient’s face Count of inflammatory/non-inflammatory lesions	X	X	X	X	X	X		
Blood tests (liver function test, renal function test)	X					X		
Blood tests		X				X		
Urine pregnancy test	X					X		
Randomization & allocation		X						
Investigational Produce		X	X	X	X			
Numeric rating scale (menstrual pain)		X		X		X	X	X
Verbal multidemensional scoring system grade	X	X		X		X	X	X
Investigator’s Static Global Assessment Scale of Facial Acne Vulgaris		X	X	X	X	X		
Numeric rating scale (acne)		X	X	X	X	X	X	X
Monitoring adverse events			X	X	X	X	X	X
Compliance check			X	X	X	X		
Blinding check						X		

#### Primary outcomes

Primary outcome will be presented as the mean percentage change in total lesion counts from baseline to the end of the treatment. Total lesion counts refer to the sum of the inflammatory and non-inflammatory lesions. Inflammatory lesions included inflammatory papules, pustules, and cysts and non-inflammatory lesions include black and white head comedones. The front and both sides of each participant’s face will be photographed on each visit using a Lumix-LX2 digital camera (Panasonic, Osaka, Japan). Two Korean medicine doctors who have been in clinical practice for more than 3 years will count the lesions independently. Discrepancies will be resolved by discussion with a third researcher.

#### Secondary outcomes

Secondary outcomes will be assessed using the numeric rating scale (NRS) for dysmenorrhea and acne vulgaris, VMSS for dysmenorrhea, investigator’s static global assessment scale of facial acne vulgaris, and laboratory tests.

##### Dysmenorrhea

The NRS and VMSS for dysmenorrhea will be evaluated at baseline, 4 weeks, 8 weeks, f/u 4 weeks, and f/u 8 weeks. The mean difference in NRS and VMSS between herbal medication treatment and placebo group will be assessed at 8 weeks. The difference in NRS and VMSS between two groups will also be assessed according to time points.

##### Acne vulgaris

The mean percentage change in inflammatory and non-inflammatory lesion counts from baseline to the end of treatment (8 weeks) will be evaluated as secondary outcomes. We will also analyze the mean difference in NRS and investigator’s static global assessment scale of facial acne vulgaris between herbal medication treatment and placebo group at 8 weeks. Furthermore, the difference in NRS and investigator’s static global assessment scale of facial acne vulgaris between two groups will be assessed every 2 weeks (0–8 weeks).

### Safety and adverse event outcomes

#### Safety

Safety will be investigated using adverse reaction reports and clinical laboratory tests. We will examine blood test results (BUN, creatinine, ALT, AST) and urine hCG levels at screening (visit 1) and 8 weeks (visit 6).

#### Adverse events

We will evaluate and classify the adverse events (AEs) at every visit throughout the trial. An AE is an undesirable, unintended sign, symptom, or disease that does not necessarily have a cause–effect relationship with the intervention evaluated in a clinical trial. We will continuously monitor AEs and make any decision in this regard on the basis of both objective and subjective signs as well as blood test results. Appropriate measures will be taken immediately to minimize any SAEs.

### Sample size calculation

We referred to similar studies that investigated the effects of herbal medicine for dysmenorrhea [17, 21]. The mean difference for the treatment group and placebo group was assumed to be 2.21 and 1.28, respectively. Pooled SD was assumed to be 2.24 based on the same studies. We set α at 0.05 and 1-β at 0.8 and calculated the necessary sample size. Assuming a 15% dropout rate and an allocation ratio of 1, the sample size was calculated to be 116 (58 participants in each group).

### Data management

Data entry and management will be completed by an independent data administrator to ensure data accuracy. All procedures comply with confidentiality standards for medical data. All data will be entered electronic case report form and all source document will be kept at the study site. Important protocol modifications during this study will be communicated to the Institutional Review Board, trial registry, investigators, trial participants and the journal of publication.

### Statistical analysis

We will analyze the data using the intent-to-treat (ITT) principle and per protocol principle. We, along with a professional statistician, will perform data analysis of the results. We will not perform the interim analysis.

#### Patient groups for data analysis

##### Intent-to-treat population

The ITT population will include all participants treated with at least one dose of the study drug. If the patients dropped out during the trial or if there is missing data, the “last observation carried forward” principle will be used to compensate for that data.

##### Per-protocol population

The per-protocol population will include patients who would consume more than 80% of the prescribed doses of the study drug and complete the trial.

Effectiveness will be evaluated by ITT principle and per-protocol principle. When analysis results are different, the ITT principle will be considered for the main analysis and subordinately, we will compare the results of the analysis based on the per-protocol principle. Demographic and safety test will be analyzed by ITT principle and the data for safety test will be used without adjustment.

#### Primary endpoint

An independent t-test (or Mann-Whitney U test) will be used to compare the mean percentage change in total lesion counts from baseline to the end of the treatment between the two groups in terms of the primary outcome. Superiority will be confirmed when there are statistically significant differences. The level of significance will be set at a *P* value of 0.1.

#### Secondary endpoint

An independent t-test (or Mann-Whitney U test) will be used for continuous variables and Chi-square test (or Fisher’s exact test) for categorical variables between groups. Group effect, time effect, and group × time effect for repeat measurement data will be analyzed by repeated-measures analysis of variance (ANOVA) or repeated-measures analysis of covariance (ANCOVA).

## Discussion

Nowadays, adult acne, especially female adult acne is increasing considerably [6].Acne is primarily associated with phychosocial impairment. It can affect an individual’s self-esteem and may cause anxiety, withdrawal, decreased ability to focus on work, depression, and interpersonal conflicts, thereby impairing overall quality of life [22–25].

Acne related to menstrual cycle is often nonresponsive to conventional therapies; thus, hormonal therapy, such as oral contraceptives, is used [15]. However, oral contraceptives have adverse effects including nausea/vomiting, breast tenderness, headache, spotting/breakthrough bleeding, edema of the lower extremities in the venous system, and with some, weight gain [15]. Due to limited treatment options, acne related to menstrual cycle is considered a challenging clinical problem. Despite clinical evidence that acne associated with menstruation abnormalities naturally improves when menstruation recovers to normal, there have only been few studies on the effects of dysmenorrhea treatment on acne vulgaris at childbearing age. This is the first protocol to investigate the efficacy of herbal medicines prescribed in dysmenorrhoea treatment on the accompanying acne. This study is a randomized, placebo-controlled, double-blind, parallel-group multicenter trial. We designed this clinical trial in accordance with the Consolidated Standards of Reporting Trials guidelines [26]. The validated evaluation tool will be used to assess the severity of acne and dysmenorrhea. Moreover, pattern identification will be used to reflect clinical field. We believe that this research will provide important evidence regarding the use of herbal medicines in the treatment of acne along with menstrual abnormalities. Moreover, a larger scale clinical trial evaluating the efficacy and safety of GBH and DJS in treating menstruation-related acne, based on this study and specific Korean patterns identified, will be possible in future.

## Abbreviations

AE: Adverse event
ALT: Alanine aminotransferase
ANCOVA: Analysis of covariance
ANOVA: Analysis of variance ANOVA
AST: Aspartate aminotransferase
BMI: Body Mass Index
BUN: Blood urea nitrogen
DJS: Dangguijagyag-san
GBH: Gyejibokryung-hwan
hCG: Human chorionic gonadotropin
ITT: Intent-to-treat
NRS: Numeric rating scale
NSAID: Nonsteroidal anti-inflammatory drug
SAE: Serious adverse event
TKM: Traditional Korean medicine
VMSS: Verbal multidimensional scoring system
